# Chronic fatigue syndrome after Giardia enteritis: clinical characteristics, disability and long-term sickness absence

**DOI:** 10.1186/1471-230X-12-13

**Published:** 2012-02-08

**Authors:** Halvor Naess, Morten Nyland, Trygve Hausken, Inghild Follestad, Harald I Nyland

**Affiliations:** 1 Institute of Clinical Medicine, Department of Neurology, and Unit for Gastroenterology, Department for Medicine, Haukeland University Hospital, N-5021 Bergen, Norway

## Abstract

**Background:**

A waterborne outbreak of Giardia lamblia gastroenteritis led to a high prevalance of long-lasting fatigue and abdominal symptoms. The aim was to describe the clinical characteristics, disability and employmentloss in a case series of patients with Chronic Fatigue Syndrome (CFS) after the infection.

**Methods:**

Patients who reported persistent fatigue, lowered functional capacity and sickness leave or delayed education after a large community outbreak of giardiasis enteritis in the city of Bergen, Norway were evaluated with the established Centers for Disease Control and Prevention criteria for CFS. Fatigue was self-rated by the Fatigue Severity Scale (FSS). Physical and mental health status and functional impairment was measured by the Medical Outcome Severity Scale-short Form-36 (SF-36). The Hospital Anxiety and Depression Scale (HADS) was used to measure co-morbid anxiety and depression. Inability to work or study because of fatigue was determined by sickness absence certified by a doctor.

**Results:**

A total of 58 (60%) out of 96 patients with long-lasting post-infectious fatigue after laboratory confirmed giardiasis were diagnosed with CFS. In all, 1262 patients had laboratory confirmed giardiasis. At the time of referral (mean illness duration 2.7 years) 16% reported improvement, 28% reported no change, and 57% reported progressive course with gradual worsening. Mean FSS score was 6.6. A distinctive pattern of impairment was documented with the SF-36. The physical functioning, vitality (energy/fatigue) and social functioning were especially reduced. Long-term sickness absence from studies and work was noted in all patients.

**Conclusion:**

After giardiasis enteritis at least 5% developed clinical characteristics and functional impairment comparable to previously described post-infectious fatigue syndrome.

## Background

Fatigue is a prominent symptom of many acute infections. In a minority of the infected individuals the period of convalescence is followed by a post-infectious illness with disabling fatigue, musculoskeletal pain and neurocognitive difficulties [1]. Both viral and non-viral micro-organisms have a causative role in triggering a chronic fatigue syndrome [2]. The functional and occupational morbidity in these patients is considerable with work-related disability reflected in long-term sickness absence from studies and employment [3,4].

Giardia lamblia infections are rare in our area. However due to contamination of the municipal water supply in Bergen, Norway, in 2004, 1262 (764 females and 498 males) laboratory-confirmed cases of Giardia lamblia were registered [5,6]. All cases received a questionnaire in 2006 including the following concerning fatigue: " Do you have problems with fatigue?" (less or same as usual/more than usual//much more than usual). The last two answer options were defined as fatigue. The response rate was 81% (1017/1262) and fatigue was reported by 41% (419/1017) as compared to 22% in the general population [7,8]. Patients with loss of employment or delayed education due to severe fatigue were admitted to a specialist chronic fatigue clinic at the Department of Neurology, Haukeland University Hospital, Bergen, Norway for clinical evaluation with Centers for Disease Control and Prevention (CDC) criteria [9-11].

The goals of the present study were to describe the clinical characteristics of CFS in this patient cohort and to assess and document disability and the level of work related impairment and loss of employment [4,9,12,13]. The course of the illness from onset of abdominal symptoms due to Giardia enteritis to the development of severe fatigue is of interest in order to evaluate of the association between acute infection and the subsequent severe fatigue. Previous studies have reported both sudden and gradual onset of illness [14-17]. The present study aims to shed some light on this issue by describing the clinical picture of CFS following a common infectious agent.

Giardia enteritis appears to have precipitated a small outbreak of CFS previously [18]. To our knowledge this is the first report of CFS after a large community outbreak of confirmed Giardia enteritis.

## Methods

### Patients

From August 2005 to September 2007, 96 patients (29 males and 67 females) with laboratory verified Giardia lamblia infection during autumn 2004 were referred to a specialist chronic fatigue clinic at the Department of Neurology, Haukeland University Hospital for clinical evaluation of severe long-lasting post-infectious fatigue with functional impairment for work or studies. The patients were either referred from general practitioners in Bergen (5 cases) or from the Department of Medicine, Haukeland University Hospital (91 cases). Primary-care records were available and examined. In addition to written self-report of past medical and psychiatric history the patients were interviewed about exclusionary medical and psychiatric diagnosis (psychosis, bipolar disorder, substance misuse, an organic brain disorder, or an eating disorder) by an experienced clinical neurologist. Patients fulfilling the CFS criteria [10] were included in the present study and underwent further evaluation as detailed below. We excluded patients with an interval free of symptoms between the giardiasis enteritis and the development of CFS.

All patients completed a checklist of symptoms for details see Table 1. At the time of referral the patients were asked to quantify abdominal symptoms (nausea, bloating, abdominal pain, constipation, and diarrhea) on a scale from 0 to 10 where 10 represented worst symptoms (Table 2). Social adversity and stressful life events and difficulties such as disease or death in close family, marital problems, financial problems, and occupational stress based on self-report were recorded [19]. A shortened Eysenck personality inventory neuroticism (EPQ-N) scale was used [20].

**Table 1:** Symptoms at the time of referral (n = 58)

	Total n (%)	Females n (%)	Males n (%)	P*
Headache	41 (71)	32 (73)	9 (64)	.74

Myalgia	40 (69)	33 (75)	7 (50)	.10

Arthralgia	30 (52)	25 (57)	5 (36)	.22

Flu-like symptoms	37 (64)	32 (73)	5 (36)	.023

Frequent sore throat	27 (47)	23 (52)	4 (29)	.14

Tender lymph nodes	11 (19)	9 (21)	2 (14)	1.00

Visual disturbances	22 (38)	18 (41)	4 (29)	.53

Dizziness	42 (72)	33 (75)	9 (64)	.50

Paresthesia	24 (41)	21 (48)	3 (21)	.12

Muscle weakness	47 (81)	37 (84)	10 (71)	.43

Photo/phonophobia	35 (60)	32 (73)	3 (21)	.001

Reduced stress tolerance	52 (91)	42 (96)	10 (77)	.072

Reduced sleep quality	37 (64)	29 (66)	8 (57)	.75

Increased need of sleep	51 (89)	39 (89)	12 (86)	1.000

Depression	23 (40)	17 (39)	6 (43)	1.000

Anxiety	21 (37)	17 (40)	4 (29)	.54

Concentration problems	56 (97)	43 (98)	13 (93)	.43

Memory problems	45 (78)	37 (84)	8 (57)	.06

Confusion	18 (32)	13 (30)	5 (39)	.74

Word finding problems	36 (63)	28 (64)	8 (62)	1.00

Palpitations	24 (41)	20 (46)	4 (29)	.36

Fainting	14 (25)	11 (25)	3 (23)	1.000

Respiratory problems	14 (25)	12 (27)	2 (15)	.48

Frequent micturition during night	25 (44)	21 (48)	4 (31)	.35

Changed temperature regulation	28 (49)	26 (59)	2 (15)	.010

Night sweating	24 (42)	18 (41)	6 (46)	.76

* Fisher's exact test comparing females and males

**Table 2:** Mean abdominal symptom severity on referral (range 0-10 (severe)) (n = 58)

	Total	Females	Males	P*
Nausea	2.8	3.2	1.9	.15

Bloating	5.2	5.7	3.6	.02

Abdominal pain	3.7	3.9	2.9	.23

Constipation	1.6	1.7	1.2	.51

Diarrea	3.8	3.9	3.5	.68

Mean abdominal complaints	3.4	3.7	2.6	.06

* Student's t-test

Fatigue was self-rated by the Fatigue Severity Scale (FSS) which consists of nine statements regarding fatigue experiences. Patients were asked whether they disagreed (1) or agreed (7) using a Likert scale (range 1 to 7). Higher scores indicate higher levels of fatigue. Patients with a mean FSS score > 5 are defined as having severe fatigue [21,22].

Time to fatigue after onset of enteritis was defined as immediately, taking weeks, or months.

To assess functional impairment we used the Short-Form Health Survey (SF-36). The SF-36 has eight subscales: physical functioning (PF), role limitations due to physical health problems (role-physical, RP), bodily pain (BP), general health (GH), vitality (VT), social functioning (SF), role limitations due to emotional health problems (role-emotional, RE) and mental health (MH). The first four sub-scales primarily measure physical health and the last four primarily measure mental health. A distinctive pattern of impairment has been reported for CFS when patients in different countries are evaluated [23]. Normative SF-36 data by gender and age are available for the general population in Norway [24].

The Hospital Anxiety and Depression Scale (HADS) was used to evaluate anxiety and/or depression (HADS-A and HADS-D subscales respectively) [25]. There are normative data from Swedish and English studies [26,27]. Inability to work or study was recorded as sickness absence certified by a doctor or delayed education [28].

An important outcome question was the patients' rating of overall worsening or improvement since the onset of their symptoms: worsening, no change, or improvement.

The study was approved by the local ethics committee.

### Statistics

Students t-test, Mann-Whitney U test, Fisher's exact test, and Pearson Chi square were used when appropriate. All analyses were preformed with SPSS 14.0 for Windows. *P *values < .05 were considered statistically significant.

## Results

A total of 96 patients were referred to the fatigue clinic at the Department of Neurology, Haukeland University Hospital with prolonged fatigue and accompanying symptoms. Fifty-eight (60%) patients, 20 students and 38 workers, fulfilled the criteria for CFS; 44 (76%) females and 14 (24%) males (*P *= .02, Fisher's exact test including all patients with Giardia lamblia confirmed infection) comprising 5% of the patients with laboratory confirmed Giardia lamblia. The mean age at onset of the disease for females was 35.3 years (SD 11.2) and for males 29.2 years (SD 11.7) (*P *= .09, Student's t-test). The mean age at the time of referral for females was 38.0 years (SD 11.3) and for males 31.7 years (SD 11.6) (*P *= .07, Student's t-test). The mean duration of the illness was 2.7 years (SD .4; range 1.5 to 3.0 years). Table 3 shows sickness absence among workers and students.

**Table 3:** Patient demographics and employment status

Demographics	
Female, n (%)	44 (76)

Age, mean, female	35.3

Age, mean, male	29.2

Education	

< college degree, n (%)	14 (24)

college degree and post graduate, n (%)	44 (76)

Employment status (n = 38)	

Working half-time, n (%)	13 (22)

Sickness absence, n (%)	22 (38)

Disability pension, n (%)	3 (5)

Education status (n = 20)	

Studies half-time, n (%)	6 (10)

Sickness absence, n (%)	14 (24)

Table 1 shows the symptoms on referral for both females and males. There were little differences between the sexes. Females reported more severe abdominal symptoms than males (*P *= .06, Fisher's exact test) (Table 2).

Depression any time before the Giardia infection was reported by 11/55 (20%) patients (mean time 10.8 years before the Giardia infection). Anxiety any time before the Giardia infection was reported by 9/55 (16%) patients (only females) (mean time 15.5 years before the Giardia infection). Table 4 shows stress episodes during the 6 months prior to the Giardia infection. There were more stressful life events prior to Giardia enteritis among patients with fatigue development over months compared to patients with fatigue development within weeks (mean 1.1 events versus mean .6 events; *P *= .15, Mann-Whitney U test).

**Table 4:** The experience of life events and difficulties during the 6 months prior to Giardia infection

	Total n (%)	Females n (%)	Males n (%)	P*
Diseases in close family	10 (18)	9 (21)	1 (8)	.43

Education difficulties	10 (18)	7 (16)	3 (23)	.68

Work difficulties	8 (14)	8 (18)	0 (0)	.18

House or car difficulties	2 (4)	1 (2)	1 (8)	.41

Death in distant family or among friends	2 (4)	2 (5)	0 (0)	1.000

Financial difficulties	5 (9)	4 (9)	1 (8)	1.000

Death in close family	6 (11)	5 (11)	1 (8)	1.000

Disease in distant family or among friends	2 (4)	2 (5)	0 (0)	1.000

Marital difficulties	4 (7)	3 (7)	1 (8)	1.000

Vacation difficulties	2 (4)	1 (2)	1 (8)	.41

* Fisher's exact test comparing females and males

Time from onset of acute Giardia infection until development of debilitating fatigue was reported as acute among 16/58 (28%), taking weeks among 8/58 (14%) and taking months among 34/58 (58%). Patients who developed debilitating fatigue over months had more initial abdominal symptoms than patients who developed debilitating fatigue within weeks of Giardia enteritis (*P *= .06, Pearson Chi square).

At the time of referral 9/58 (16%) patients reported improvement, 16/58 (28%) reported no change, and 33/58 (57%) reported slight or significant worsening. The mean fatigue score for FSS was 6.6 (SD .45). The mean FSS score for females was 6.64 (SD .45) and for males 6.44 (SD .43) (*P *= .15, Student's t-test).

**Figure 1:**
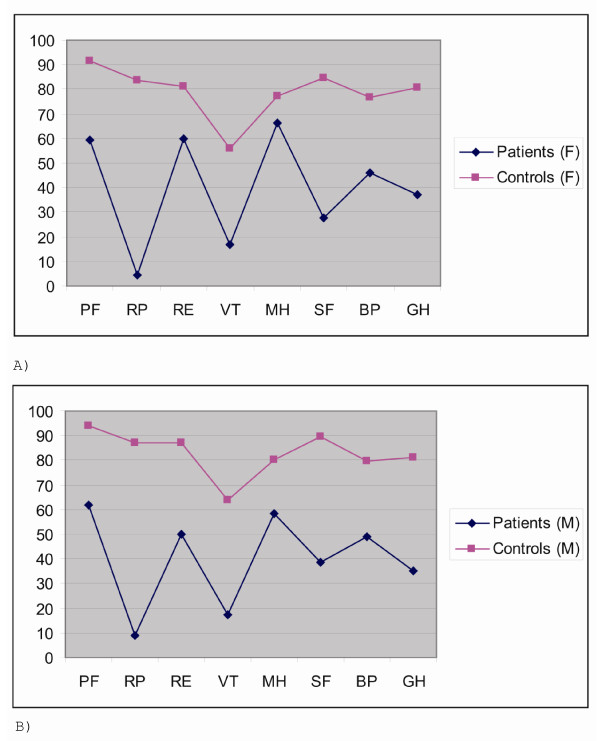
**a. Mean SF-36 subscores among female patiens on follow-up and female controls**. **b**. Mean SF-36 subscores among male patiens on follow-up and male controls. All *P *< .0001, Mann-Whitney U test.

Figure 1a, b show SF-36 subscale scores among patients and controls for both sexes (n = 58). All subscale scores were significantly reduced among patients compared to controls for both sexes (all *P *< .001, Mann-Whitney U test). The lowest scores were observed for physical capacity, vitality and social functioning while scores for emotional and mental functioning were least reduced.

There were no differences between male and female as to HADS-A (n = 58) sub-score, (7.4 versus 6.7, *P *= .58, Student's t-test) or HADS-D (n = 57) sub-score (7.1 versus 7.5, *P *= .75, Student's t-test). Compared to normative data from Sweden the patients scored higher for the HADS-A sub-score (6.9 versus 5.3, *P *= .009, Student's t-test) and for the HADS-D sub-score (7.4 versus 3.0, *P *< .001, Student's t-test)[26]. The HADS results show that 11 (22%) of the patients rated themselves at or beyond the threshold of depression (HADS-D ≥ 11) and 13 (26%) of the patients for anxiety (HADS-A ≥ 11)[25].

Mean EPQ-N for males was 4.6 (SD 3.0) and for females 5.1 (SD 2.8). These results did not differ from normative data (*P *> .25, Student's t-test) [20].

## Discussion

Our study show that at least 5% of the patients with confirmed Giardia enteritis reported failure to recover with unexplained fatigue and accompanying symptoms that in the broad corresponded with a clinical entity described previously [9,10]. In comparison, the prevalence of CFS in a normal population ranges between 0.23% and 0.56% in different populations [29,30]. Thus, the frequency of CFS among patients with confirmed Giardia infection was at least 8 times higher than in the general population. Our findings suggest that there may be a relationship between Giardia infection and CFS. However, further studies are needed to prove that there is a causative relationship. Persisting abdominal complaints classified as diarrhoea-predominant IBS were present in 10% of the patients with confirmed Giardia infection [6]. Similar prevalence rates of 5% with CFS and 10% with IBS have been observed in patients after campylobacter gastroenteritis [31]. The rate of post-infectious fatigue syndrome after glandular fever has been reported to be 9% in previous cohort studies [2,32].

The patients in our cohort represent young adults not exposed to Giardia lamblia before the contamination of the municipal water in 2004. Although Giardia lamblia is not an invasive parasite, proteins on the surface induce a humoral immune reaction [33] and CFS pathogenesis is likely to include immunologic components. However, the relationship between inflammatory responses and post-infectious fatigue syndrome have not been documented in longitudinal studies [2].

In the present study the frequency of CFS was significantly higher among females. Other studies have also reported that CFS is more frequent among females [9,34]. By contrast, there was no gender difference as to the frequency of self-reported chronic fatigue two years after disease onset among the laboratory confirmed cases of Giardia lamblia in Bergen in accordance with previous community-based studies of chronic fatigue [8].

There were little differences between the sexes both regarding concomitant symptoms such as abdominal symptoms and other symptoms in the early phase of the disease. However, at the time of referral females reported more severe abdominal symptoms (marginally significant).

Although the cause of CFS is unknown it is generally thought that post-infectious fatigue develops shortly after acute infection [35,36]. However, more than half of our patients had a gradual onset of fatigue. Other studies have shown conflicting results as to onset of symptoms [9,37]. We found that patients who developed fatigue over months, tended to have more initial abdominal symptoms than patients who developed fatigue within weeks of Giardia enteritis. A possible explanation is that many initial symptoms either masked the experience of early fatigue or the recall of early fatigue among some patients. This may be of special importance in cases of litigation where the timing of fatigue onset after acute infection may determine the question of causation.

There was a slight trend towards more stressful life events prior to Giardia enteritis among patients with fatigue development over months compared to patients with fatigue development within weeks [19]. A possible interpretation is that whereas early onset of fatigue is caused by the Giardia enteritis, late onset of fatigue among some patients has more complex causation including psychologically stressful life events present before the Giardia enteritis [35].

The patients scored significantly worse than controls on all subscale scores of SF-36 for both sexes. The scores were lowest for physical functioning and vitality whereas emotional and mental scores were only mildly impaired. The scores among our patients were remarkably similar to the scores reported among patients with CFS in a previous study [23]. This suggests a similar disease profile among our patients and patients with CFS not related to Giardia enteritis and provides evidence that CFS exists as a discrete illness characterised by extremely low levels of physical functioning and only mild mental and emotional components [15,38,39].

The patients scored significantly worse than the controls both as to anxiety and depression based on the HADS subscores [26]. There were no sex differences between our patients as to HADS subscores. This is surprising because no males reported anxiety any time prior to disease onset while this was reported by 9 (21%) females. The CFS sufferers are not primarily depressed, and do not exhibit illness behaviour [40]. Based on the inclusion criteria which included primary-care records, the frequencies of depression and anxiety among our patients based on HADS subscores do not represent primary depression or anxiety, but reflect co-morbid illness secondary to long-standing chronic fatigue. Others have found similar or higher HADS subscores among patients with CFS [3,31,41]. Our patients did not disclose any abnormal neuroticism.

CFS is associated with various functional limitations both for work and social life, and assessment of functional ability including sickness abscence is necessary in medical and vocational rehabilitation [42]. The focus on function ability represents a shift in attention from symptoms to resources, possibilities and coping. After the initial evaluation our patients entered a comprehensive multidisciplinary intervention program. The program was individualized and included education focusing on psychology and coping. Rehabilitation included physiotherapy, manageable exercise program, and occupational therapy. We plan to publish a five year follow-up study of the patients.

There has been one previous report of community outbreak of CFS possibly precipitated by Giardia enteritis comprising 11 patients [18]. The mean age was 31 years which is similar to the mean age in the present study.

A limitation of the present study is that no systematic search for CFS among all patients with confirmed Giardia lamlia enteritis was performed. Thus, our findings represent a lower limit for the frequency of CFS after Giardia lamblia enteritis in a previously Giardia naïve population. Another limitation is that we compared our patients to published normative data and not a matched control group recruited from Bergen. Data on diseases and stressful events before the Giardia lamlia infection were based on self-report and therefore liable to failure of recall.

## Conclusion

In conclusion, among young, healthy adults never exposed to Giardia lamblia a significant proportion developed long-term chronic fatigue syndrome with high rates of occupational disability. There is need for the development of comprehensive rehabilitation programs.

## Competing interests

The authors declare that they have no competing interests.

## Authors' contributions

HN drafted the manuscript and performed the statistical analyses. MN participated in functional assessment of the patients and drafting of the manuscript. TH contributed to the drafting of the manuscript. IF participated in functional assessment of the patients and drafting of the manuscript. HIN conceived of the study, collected data, and contributed to the drafting of the manuscript. All authors read an approved the final manuscript.

## Pre-publication history

The pre-publication history for this paper can be accessed here:

http://www.biomedcentral.com/1471-230X/12/13/prepub
